# Whole-brain inputs and outputs of Phox2b and GABAergic neurons in the nucleus tractus solitarii

**DOI:** 10.3389/fnins.2024.1427384

**Published:** 2024-06-14

**Authors:** Liuqi Shao, Fanrao Kong, Xiaochen Tian, Tianjiao Deng, Yakun Wang, Yake Ji, Xiaoyi Wang, Hongxiao Yu, Fang Yuan, Congrui Fu, Sheng Wang

**Affiliations:** ^1^Department of Neurobiology, Hebei Medical University, Shijiazhuang, Hebei, China; ^2^Department of Sleep Medicine, Third Hospital of Hebei Medical University, Shijiazhuang, Hebei, China; ^3^Nursing School, Hebei Medical University, Shijiazhuang, Hebei, China; ^4^Hebei Key Laboratory of Neurophysiology, Shijiazhuang, Hebei, China

**Keywords:** nucleus tractus solitarii, breathing, neural circuit, neural tracing, modified rabies

## Abstract

The nucleus tractus solitarii (NTS) plays a critical role in the homeostatic regulation of respiration, blood pressure, sodium consumption and metabolic processes. Despite their significance, the circuitry mechanisms facilitating these diverse physiological functions remain incompletely understood. In this study, we present a whole-brain mapping of both the afferent and efferent connections of Phox2b-expressing and GABAergic neurons within the NTS. Our findings reveal that these neuronal populations not only receive monosynaptic inputs primarily from the medulla oblongata, pons, midbrain, supra-midbrain and cortical areas, but also mutually project their axons to these same locales. Moreover, intense monosynaptic inputs are received from the central amygdala, the paraventricular nucleus of the hypothalamus, the parasubthalamic nucleus and the intermediate reticular nucleus, along with brainstem nuclei explicitly engaged in respiratory regulation. In contrast, both neuronal groups extensively innervate brainstem nuclei associated with respiratory functions, although their projections to regions above the midbrain are comparatively limited. These anatomical findings provide a foundational platform for delineating an anatomical framework essential for dissecting the specific functional mechanisms of these circuits.

## 1 Introduction

The nucleus tractus solitarii (NTS) plays an important role in regulation of numerous physiological processes, such as homeostatic control of breathing (Fu et al., 2017, 2019; Yu et al., 2022; Wang et al., 2024), blood pressure (Jun et al., 2023), energy balance (Cheng et al., 2020), inflammation-induced anxiety (Brunet and Pattyn, 2002; Zhao et al., 2024), sodium appetite (Geerling et al., 2006; Resch et al., 2017) and nausea-associated behaviors (Zhang et al., 2020). These differential processes require to recruit different subpopulation of NTS neurons and the associated distinct neural circuits. For instances, cholecystokinin and dopamine β-hydroxylase (DβH) neurons in the NTS directly contribute to regulation of anorexia (Roman et al., 2016); NTS catecholaminergic neurons mediate hypoglycemic hunger via medial hypothalamic feeding pathways (Aklan et al., 2020); Calcitonin receptor-residing NTS neurons mediate food intake suppression (Cheng et al., 2020). Paired-like homeobox 2b (Phox2b), as a transcription factor, is expressed in and essential for the development of the reflex circuits that control bodily homeostasis by regulating cardiovascular, respiratory and digestive functions (Brunet and Pattyn, 2002; Dubreuil et al., 2008). Recently, we demonstrate that a subset of Phox2b-expressing NTS (NTS^Phox2b^) neurons serves as central respiratory chemoreceptors to maintain ventilatory homeostasis, gaining better understanding of neural control of breathing by this subpopulation. Moreover, activation of both leptin receptor b and orexin 1 receptor-expressing NTS neurons facilitates breathing (Yu et al., 2022; Wang et al., 2024), further highlighting molecular identify.

To unveil the function-specific neural circuits, it is necessary to determine the afferent and efferent projections of NTS neurons. It is well-established that glutamatergic, catecholaminergic and GABAergic neurons, accounting for the majority of subtypes in the NTS, have been observed to regulate multiple physiological functions. To date, a few studies have demonstrated neuroanatomical connections of NTS neurons with other brain regions. Neural tracing data from Loewy laboratory revealed anatomical and functional connections of the enzyme 11-beta-hydroxysteroid dehydrogenase type 2-expressing NTS neurons (Geerling and Loewy, 2006; Resch et al., 2017; Gasparini et al., 2019). Subsequently, Gasparini and colleagues utilized an axonal tracer (cholera toxin b) to provide a brain-wide map of neurons that extend their axons to the caudal NTS (Gasparini et al., 2020). Recently, we characterized whole-brain monosynaptic inputs and outputs of leptin receptor b-expressing neurons within the NTS (Sun et al., 2023), providing a neuroanatomical basis for further research on physiological and pathological functions of these neurons. NTS^Phox2b^ neurons represent the majority of excitatory neurons, including glutamatergic and catecholaminergic, whereas Phox2b was almost absent in GABAergic NTS (NTS^GABA^) neurons (Kang et al., 2007). GABAergic neurons are abundant in the NTS, with a characteristic of both short- and long-range projections (Shi et al., 2021). Nevertheless, studies on connections linking both NTS^Phox2b^ and NTS^GABA^ neurons with other brain regions are required to provide a comprehensive understanding of function-specific neural circuits of these neurons.

In the present studies, we employed transsynaptic retrograde rabies virus and common anterograde virus-based tracing strategies to provide a detailed whole-brain mapping of afferent and efferent projections of both NTS^Phox2b^ and NTS^GABA^ neurons. Building on previous neural tracing evidence, we extended the investigation to concurrently visualize efferent pattern of both excitatory and inhibitory NTS neurons from Phox2b-Flop::Vgat-Cre mice. These data provide a foundational anatomical framework for future investigations into the interregional pathways that regulate NTS functions.

## 2 Materials and methods

### 2.1 Animal

Adult Phox2b-Cre (IMSR Cat# JAX: 016223), Phox2b-Flpo (IMSR Cat# JAX: 022407) and Vgat-Cre (IMSR Cat# JAX: 016962) mice of either sex used in the experiments were purchased from the Jackson Laboratory. Phox2b-Cre (Scott et al., 2011) and Phox2b-Flpo (Hirsch et al., 2013) mice have Cre or Flpo recombinase expression under control of the Phox2b promoter/enhancer regions within the BAC transgenic mouse line. Phox2b-Flpo::Vgat-Cre mice were generated by crossing Vgat-Cre mice with Phox2b-Flpo mice. Mice were housed under program-controlled temperature (23 ± 1°C) and humidity (50% ± 10%) with a fixed 12 h light/12 h dark cycle and with *ad libitum* access to food and water. All experiments were performed in accordance with the Guide for the Care and Use of Laboratory Animals, and were approved by the Animal Care and Ethics Committee of Hebei Medical University.

### 2.2 Viruses

AAV2/9-EF1α-DIO-EGFP-T2A-TVA (2.0 × 10^12^ genome copies/mL), AAV-EF1α-DIO-N2cG (2.0 × 10^12^ genome copies/mL) and RV-EnvA-∆G-tdTomato (1.0 × 10^8^ genome copies/mL) were purchased from BrainCase (Wuhan, China). The two Cre-dependent AAVs were mixed at a 1:1 ratio as helper viruses for retrograde monosynaptic-tracing experiments. AAV2/9-EF1a-DIO-EGFP (5.04 × 10^12^ genome copies/mL) and AAV2/9-EF1a-fDIO-mCherry (6.0 × 10^12^ genome copies/mL) were used for anterograde tracing experiments and was packaged by BrainVTA (Wuhan, China).

### 2.3 Surgery and viral injections

Surgical procedures were conducted in accordance with methodologies outlined in prior studies (Sun et al., 2023). Adult mice underwent anesthesia induced by intraperitoneal administration of pentobarbital sodium at a dosage of 60 μg/g. Anesthetic depth was monitored by the absence of corneal and hindpaw withdrawal reflexes, with supplemental anesthetic provided as needed, amounting to 30% of the initial dosage. All procedures were performed under sterile conditions. Post-anesthesia, the mice were positioned prone in a stereotaxic frame (RWD Life Science Co., Ltd., Shenzhen, China) and body temperature was maintained at 37°C using a heating pad and a blanket. Viral vectors were delivered precisely to the NTS at the level of the calamus scriptorius (anterior–posterior (AP): + 0.2 mm, medial-lateral (ML): ± 0.3 mm; dorsal-ventral (DV): − 0.2 mm; AP: + 0.3 mm, ML: ± 0.4 mm, DV: − 0.3 mm) using a glass micropipette. For retrograde tracing, injections of helper viruses (100 nL per injection) were administered unilaterally into the NTS and were allowed to diffuse for 10 min. T Following a two-week interval, RV-EnvA-∆G-tdTomato (160 nL) was injected into the same site. Mice were perfused for immunostaining 1 week later.

In the case of anterograde tracing, we unilaterally injected AAV-EF1α-DIO-EYFP into the NTS of Phox2b-Flpo::Vgat-Cre mice (two injections, 80 nL per injection). After 2 weeks, a second series of injections involving AAV-EF1α-fDIO-mCherry (80 nL per injection) were administrated at the same locations in the same subjects. Post-injection, the mice were treated with the antibiotic ampicillin (125 mg/kg, i.p.) and the analgesic ketorolac (4 mg/kg, i.p.). A 4-week recuperation period was observed before conducting histological analyses.

### 2.4 Histology and immunostaining

Mice were subjected to deep anesthesia using urethane (1.8 g/kg, intraperitoneally) and subsequently perfused transcardially with saline, followed by fixation with 4% paraformaldehyde in phosphate-buffered saline (PBS). Post-fixation, brains were excised on ice and preserved in 4% paraformaldehyde at 4°C for 12 h, after which they were transferred into 30% sucrose solution buffered with PBS for cryoprotection. Coronal sections (25 μm) were harvested on a freezing microtome (CM1950; Leica Microsystems, Germany). For immunohistochemistry, sections were initially blocked using 5% bovine serum albumin in PBS containing 0.25% Triton X-100 for 30 min at room temperature. This step was followed by overnight incubation at 4°C with primary antibodies diluted in PBS supplemented with 2% bovine serum albumin. Sections were subsequently washed thrice in PBS for 5 min each and incubated with fluorescently-labeled secondary antibodies for 2 h at room temperature. All immersion steps and incubations were conducted on a shaker set to a gentle speed. After a final series of washes in PBS, sections were mounted on glass slides using Vectashield Antifade Mounting Medium (Vector Laboratories, Burlingame, CA, United States) to enhance fluorescence retention and prevent photobleaching during microscopic examination.

The primary antibodies used were as follows: chicken anti-EGFP (dilution 1:1000, Abcam, catalog# ab13970, RRID: AB_300798), rabbit anti-mCherry (dilution 1:1000, Novus Biologicals, catalog# NBP2-25157, RRID: AB_2753204). The fluorophore-conjugated secondary antibodies used were: Alexa Fluor^®^ 488 Goat Anti-Chicken IgY (H + L) (dilution 1:1000, Abcam, catalog# ab150169, RRID: AB_2636803), Cy™3 AffiniPure™ Goat anti-Rabbit IgG (H + L) (dilution 1:1000, Jackson ImmunoResearch Laboratories, catalog# 111-165-003, RRID: AB_2338000).

### 2.5 Imaging and data analysis

Images of whole-brain sections were acquired using a laser scanning confocal microscope with either 10x or 20x objectives, and detailed magnification was achieved through the employment of a 20x objective on a Zeiss microscope (LSM 900; Carl Zeiss, Jena, Germany). The delimitation of subregional boundaries was referenced against the Mouse Brain Atlas (Paxinos and Franklin, 2013). Quantitative analysis involved the manual enumeration of tdTomato-expressing neurons within distinct nuclei. The relative prevalence of input neurons across 86 brain regions was determined by calculating the ratio of afferent cells in each nucleus to the total population of tdTomato-marked cells, excluding those at the injection sites. Neuronal distributions across each brain region accounted for at least 0.05% of the total monosynaptic inputs were specifically noted.

For the quantification of whole-brain efferent projections emanating from NTS^Phox2b^ and NTS^GABA^ neurons, analyses focused on the proportion of axonal projections, computed as the ratio between the average count of axonal varicosities and the total number of varicosities observed in whole-brain projections. Further, axonal varicosities were documented using a 20x objective on the Zeiss confocal system, and the enumeration of these structures across the brain was performed semi-automatically using a particle analyzing plugin in ImageJ software. Results and statistical analyses were restricted to data derived exclusively from mice with accurately targeted injection sites, as discerned in the accompanying figures.

### 2.6 Quantification and statistical analysis

Statistical analyses were performed using the Prism software version 9 (GraphPad, La Jolla, CA). All data are presented as the mean ± SEM (standard error of the mean).

## 3 Results

### 3.1 Experimental strategy to target NTS^Phox2b^ and NTS^GABA^ neurons

**Figure 1:**
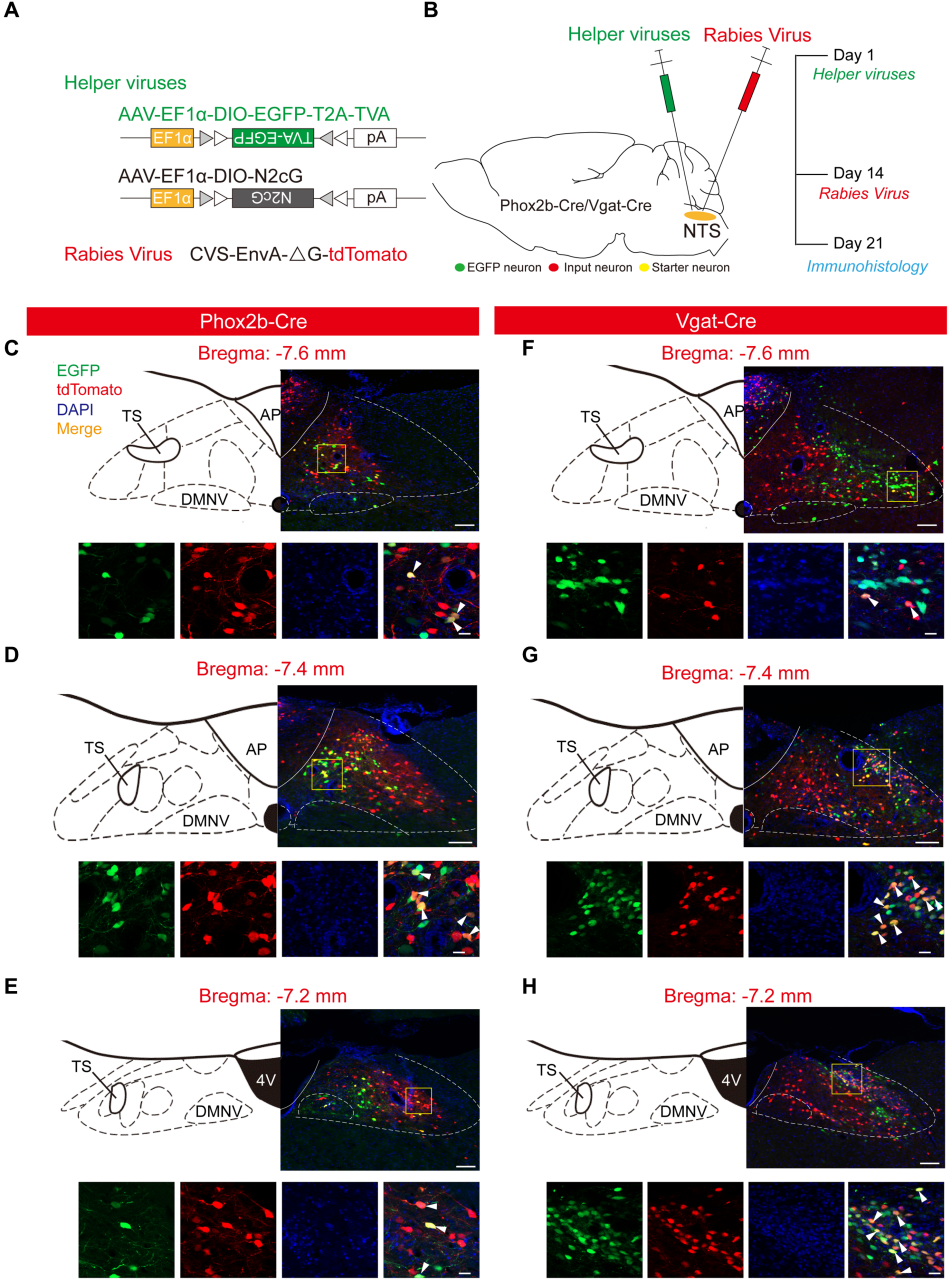
Experimental strategy to target NTS^Phox2b^ and NTS^GABA^ Neurons. **(A)** Schematic of viral vectors for RV-based trans-synaptic retrograde tracing, including helper viruses with Cre-dependent expression of TVA receptor (AAV-EF1α-DIO-EGFP-T2A-TVA) and N2cG (AAV-EF1α-DIO-N2cG). The RV was genetically modified by pseudotyping with EnvA (CVS-EnvA-∆G-tdTomato). **(B)** Schematic of viral injection strategy and experimental procedure performed in Phox2b-Cre and Vgat-Cre mice. **(C–H)** Photomicrograph showing EGFP-expressing NTS^Phox2b^ (left) and NTS^GABA^ (right) neurons (green), tdTomato-expressing input neurons (red) and starter neurons (yellow). In each panel, the bottom images are the enlarged view of the yellow boxed region in the top images. Bottom images (from left to right): EGFP, tdTomato, DAPI and composite merge. Scale bars: 100 μm (top images in each panel), 20 μm (bottom images in bottom panel).

To identify the whole-brain monosynaptic inputs onto NTS^Phox2b^ and NTS^GABA^ neurons, we utilized trans-synaptic retrograde labeling with a modified rabies virus as depicted before (Wall et al., 2010; Osakada and Callaway, 2013). This genetic approach entails two stages. Initially, the Cre-dependent helper viruses, including AAV-EF1α-DIO-EGFP-T2A-TVA and AAV-EF1α-DIO-N2cG, were unilaterally injected into the NTS from Phox2b-Cre (*n* = 3 mice) and Vgat-Cre mice (*n* = 3 mice), respectively. After 2 weeks, the modified rabies virus RV-EnvA-∆G-tdTomato was administered at the same injection site, targeting selectively infected TVA-expressing cells and transporting retrogradely into presynaptic cells. The mice were perfused 1 week after rabies virus injection, ensuring enough time for the rabies virus to retrogradely infect and express tdTomato sufficiently in the input neurons (Figures 1A,B). Since the rabies virus cannot infect cells in the absence of the TVA receptor, infection is limited to Cre^+^ cells expressing the helper AAV. Starter neurons was identified based on co-expression of AAV helper tag (EGFP) and RV-tdTomato around the injection site, and the input neurons were detected by the expression of tdTomato that directly input to NTS^Phox2b^ and NTS^GABA^ neurons. Through mapping the starter neurons in the NTS across three mouse coronal sections, we observed that they were primarily distributed in rostrocaudal position spanning from bregma −7.6 mm to −7.2 mm. In addition, we identified tdTomato-positive neurons in the NTS that do not express EGFP, indicating the presence of direct monosynaptic inputs to the NTS neurons (Figures 1C–H). However, using the same virus strategy in wild-type mice, we detected no neurons expressing EGFP or tdTomato in the NTS (*n* = 3, data not shown), demonstrating the reliability of this technique. Here we did not check peripherally monosynaptic inputs to both population of NTS neurons.

### 3.2 Whole-brain input patterns for NTS^Phox2b^ and NTS^GABA^ neurons

**Figure 2:**
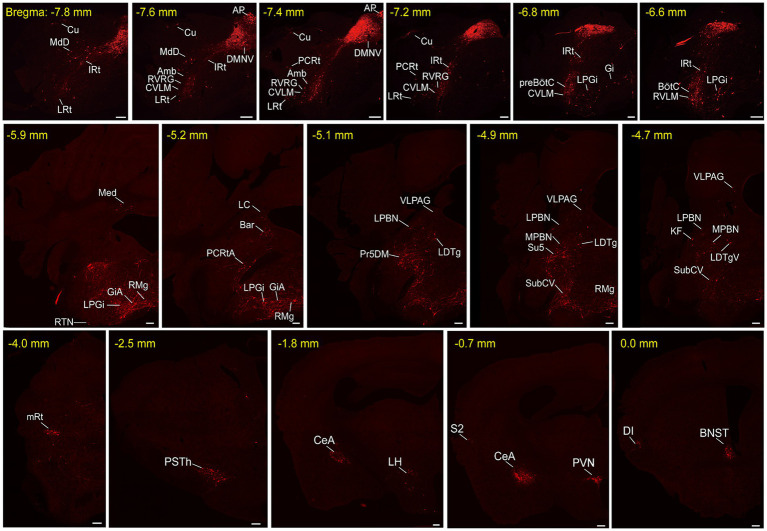
Representative images of monosynaptic inputs to NTS^Phox2b^ neurons. Input neurons of NTS^Phox2b^ neurons were identified by expression of the tdTomato. Each brain region containing these input neurons were annotated in accordance with the Mouse Brain Atlas for precise localization and identification. Scale bars, 200 μm. Cu, cuneate nucleus; MdD, medullary reticular nucleus, dorsal part; IRt, intermediate reticular nucleus; LRt, lateral reticular nucleus; AP, area postrema; DMNV, dorsal nucleus of vagus nerve; Amb, ambiguus nucleus; RVRG, rostral ventral respiratory group; CVLM, caudal ventrolateral medulla; PCRt, parvicellular reticular nucleus; preBötC, pre-Botzinger complex; Gi, gigantocellular reticular nucleus; LPGi, lateral paragigantocellular nucleus; RTN, retrotrapezoid nucleus; BötC, Bötzinger complex; RVLM, rostral ventrolateral medulla; Med, medial cerebellar nucleus; RMg, raphe magnus nucleus; GiA, gigantocellular reticular nucleus, alpha; RPa, raphe pallidus nucleus; 7 N, facial nucleus; Pr, prepositus nucleus; MVeMC, medial vestibular nucleus, magnocellular; DMSp5, dorsomedial spinal trigeminal nucleus; LC, locus coeruleus; Bar, Barrington’s nucleus; PCRtA, parvicellular reticular nucleus, alpha part; VLPAG, ventrolateral periaqueductal gray; LPBN, lateral parabrachial nucleus; Pr5DM, principal sensory trigeminal nucleus, dorsomedial part; LDTg, laterodorsal tegmental nucleus; MPBN, medial parabrachial nucleus; Su5, supratrigeminal nucleus; SubCV, subcoeruleus nucleus, ventral part; KF, Kolliker-Fuse nucleus; LDTgV, laterodorsal tegmental nucleus, ventral part; mRt, mesencephalic reticular formation; PSTh, parasubthalamic nucleus; CeA, central amygdaloid nucleus; LH, lateral hypothalamic area; PVN, paraventricular hypothalamic nucleus; S2, secondary somatosensory cortex; DI, dysgranular insular cortex; BNST, bed nucleus of the stria terminalis.

**Figure 3:**
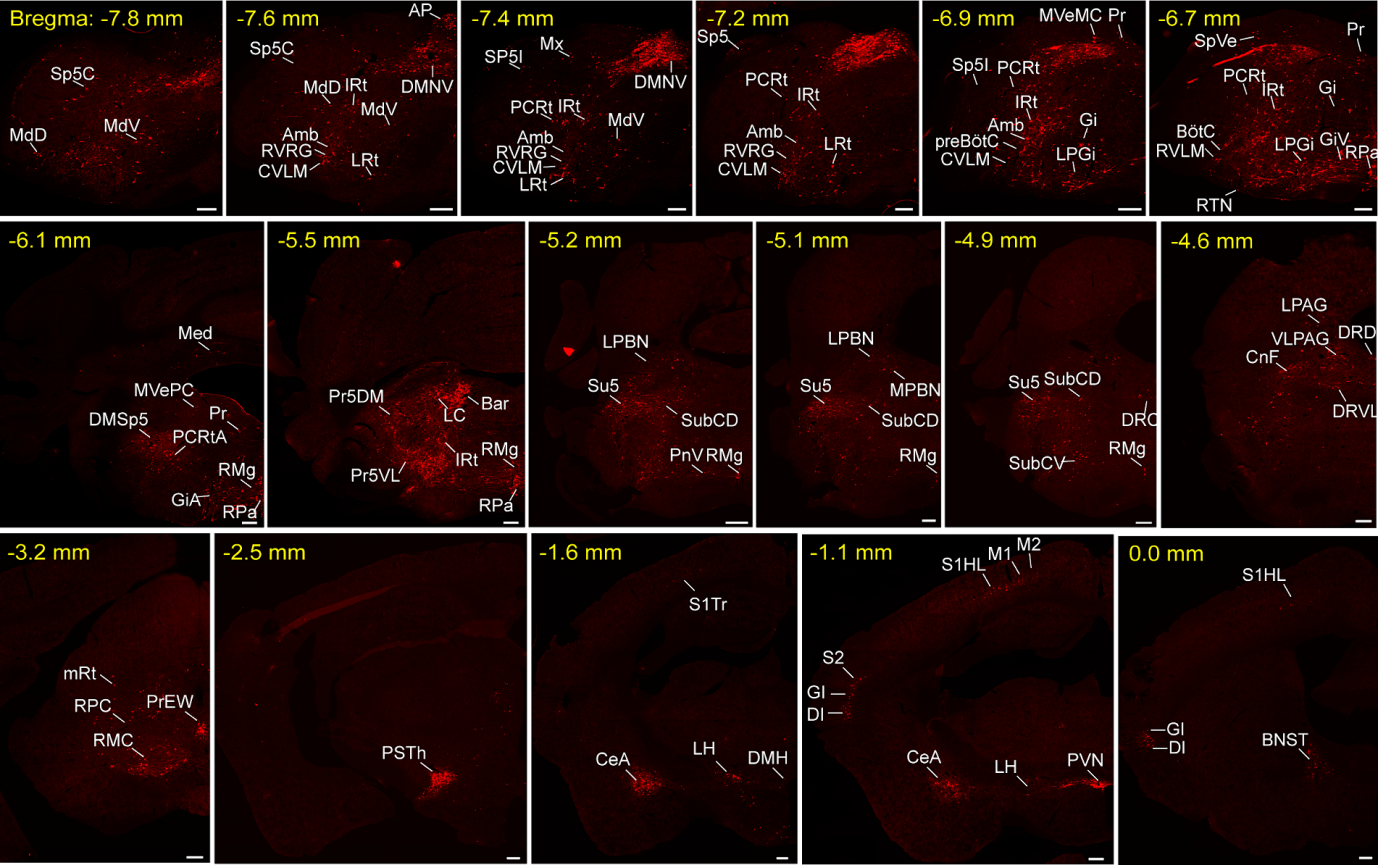
Typical images of monosynaptic inputs to NTS^GABA^ neurons. Input neurons of NTS^GABA^ neurons were characterized based on the expression of tdTomato. The brain regions housing these identified input neurons were denoted in accordance with the Mouse Brain Atlas for precise localization and identification. Scale bars, 200 μm. Sp5C, spinal trigeminal nucleus, caudal part; Cu, cuneate nucleus; MdD, medullary reticular nucleus, dorsal part; MdV, medullary reticular nucleus, ventral part; IRt, intermediate reticular nucleus; LRt, lateral reticular nucleus; AP, area postrema; DMNV, dorsal nucleus of vagus nerve; Amb, ambiguus nucleus; RVRG, rostral ventral respiratory group; CVLM, caudal ventrolateral medulla; PCRt, parvicellular reticular nucleus; Mx, matrix region of the medulla; Sp5I, spinal trigeminal nucleus, interpolar part; preBötC, pre-Bötzinger complex; SpVe, spinal vestibular nucleus; Gi, gigantocellular reticular nucleus; LPGi, lateral paragigantocellular nucleus; Pr, prepositus nucleus; GiV, gigantocellular reticular nucleus, ventral part; RTN, retrotrapezoid nucleus; BötC, Bötzinger complex; RVLM, rostral ventrolateral medulla; RPa, raphe pallidus nucleus; Med, medial cerebellar nucleus; MVePC, medial vestibular nucleus, parvicellular; DMSp5, dorsomedial spinal trigeminal nucleus; RMg, raphe magnus nucleus; GiA, gigantocellular reticular nucleus, alpha; RPa, raphe pallidus nucleus; 7 N, facial nucleus; Pr, prepositus nucleus; MVeMC, medial vestibular nucleus, magnocellular; DMSp5, dorsomedial spinal trigeminal nucleus; LC, locus coeruleus; Bar, Barrington’s nucleus; PCRtA, parvicellular reticular nucleus, alpha part; VLPAG, ventrolateral periaqueductal gray; LPBN, lateral parabrachial nucleus; Pr5DM, principal sensory trigeminal nucleus, dorsomedial part; LDTg, laterodorsal tegmental nucleus; MPBN, medial parabrachial nucleus; Su5, supratrigeminal nucleus; PnV, pontine reticular nucleus, ventral part; SubCV, subcoeruleus nucleus, ventral part; KF, Kolliker-Fuse nucleus; LDTgV, laterodorsal tegmental nucleus, ventral part; mRt, mesencephalic reticular formation; CnF, cuneiform nucleus; PrEW, pre-Edinger-Westphal nucleus; RPC, red nucleus, parvicellular part; RMC, red nucleus, magnocellular part; PSTh, parasubthalamic nucleus; S1, primary somatosensory cortex; M1, primary motor cortex; M2, secondary motor cortex; CeA, central amygdaloid nucleus; LH, lateral hypothalamic area; PVN, paraventricular hypothalamic nucleus; S2, secondary somatosensory cortex; DI, dysgranular insular cortex; GI, granular insular cortex; BNST, bed nucleus of the stria terminalis.

To unravel the monosynaptic inputs to NTS^Phox2b^ throughout the entire brain, we examined these input neurons in coronal sections from Phox2b-Cre mice based on the above neural tracing strategy. The neurons exclusively labeled with tdTomato were identified as the monosynaptic inputs to the NTS^Phox2b^ neurons. Examination of representative sections demonstrated that tdTomato-positive neurons were distributed in many brain regions, encompassing the cortex, supra-midbrain, midbrain, pons, medulla oblongata and cerebellum (Figure 2). Using the same strategy in Vgat-Cre mice, tdTomato-labeled neurons were detected in almost the same regions as identified in Phox2b-Cre mice (Figure 3).

**Figure 4:**
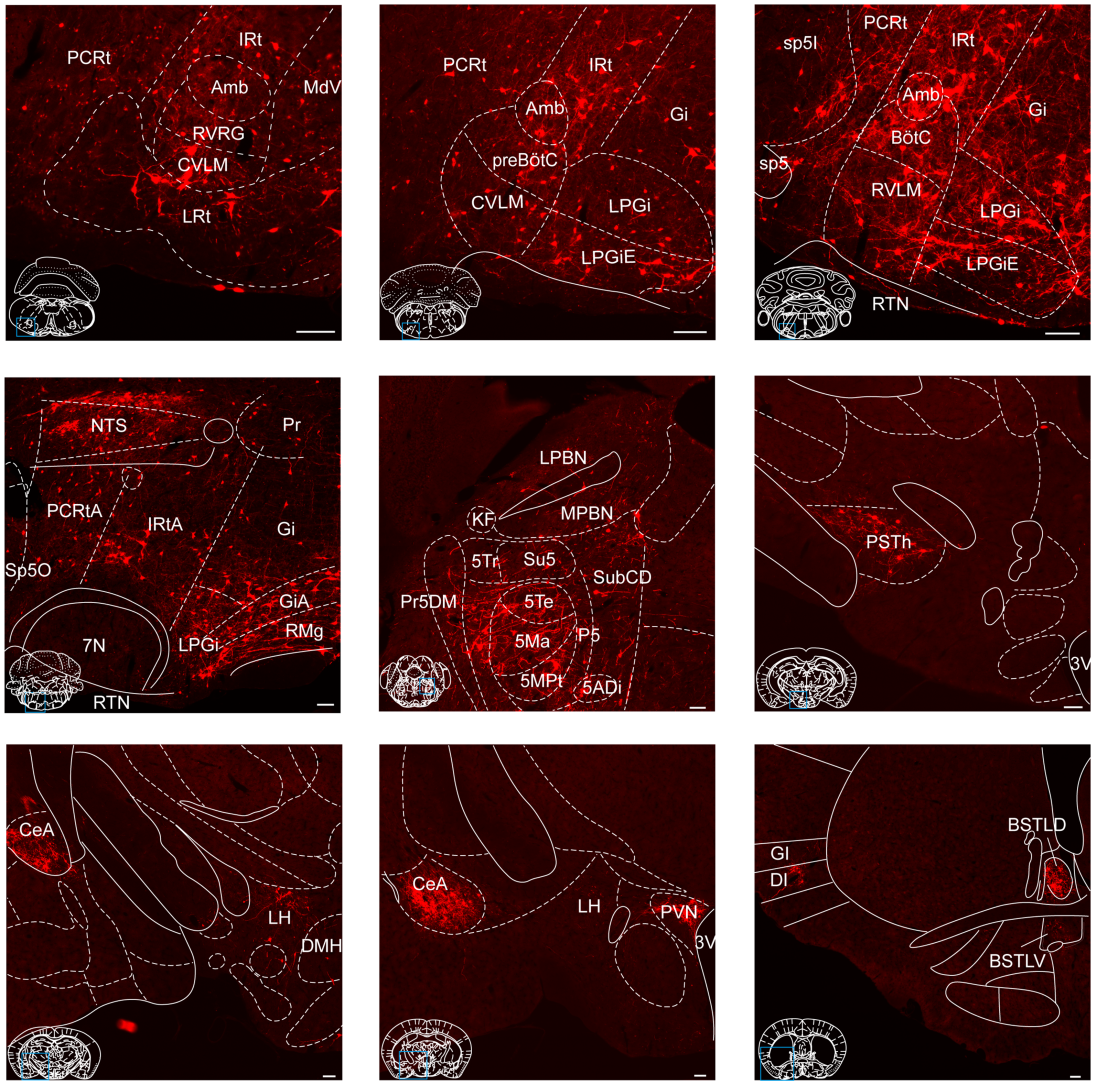
Photomicrographs of brain regions displaying dense monosynaptic inputs to NTS^Phox2b^ neurons. A larger number of input neurons targeting NTS^Phox2b^ neurons was observed in many brain regions. Scale bars, 100 μm.

**Figure 5:**
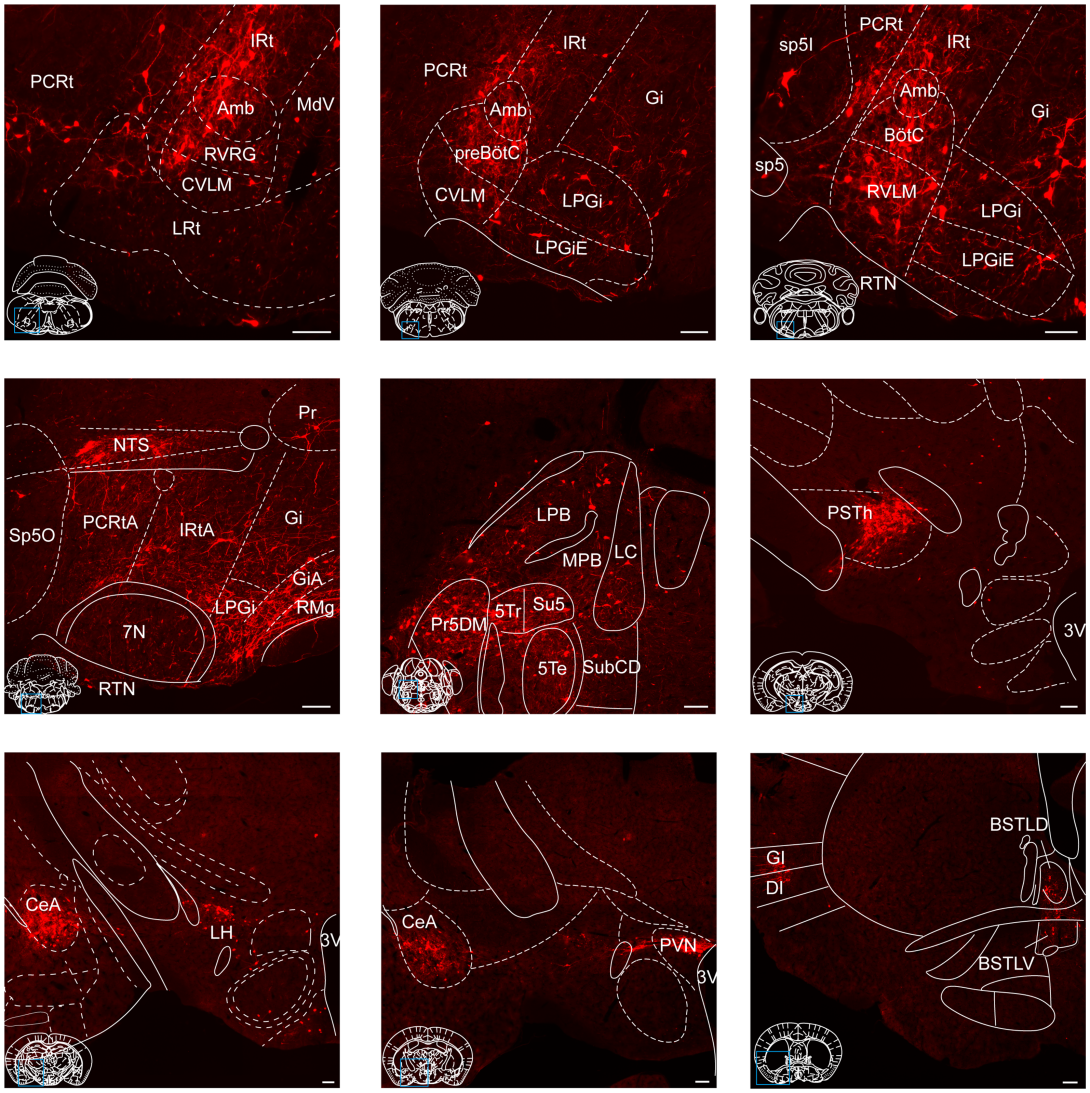
Photomicrographs of brain regions exhibiting a high density of monosynaptic inputs targeting NTS^GABA^ neurons. A larger number of input neurons innervating NTS^GABA^ neurons was found in many brain regions. Scale bars, 100 μm.

To provide a comprehensive visualization of the whole-brain distribution pattern of tdTomato-labeled input neurons to NTS^Phox2b^ (Figure 4) and NTS^GABA^ neurons (Figure 5), respectively, we selectively enlarged representative coronal images from the following crucial afferent nuclei, including the intermediate reticular nucleus (IRt), caudal ventrolateral medulla (CVLM), preBötzinger complex (preBötC), Bötzinger complex (BötC), lateral paragigantocellular nucleus (LPGi), rostral ventrolateral medulla (RVLM), ambiguus nucleus (Amb), locus coeruleus (LC), lateral parabrachial nucleus (LPBN), gigantocellular reticular nucleus (Gi), raphe magnus nucleus (RMg), parasubthalamic nucleus (PSTh), central amygdala (CeA), paraventricular hypothalamic nucleus (PVN) and lateral hypothalamic area (LH).

Additionally, these representative observations indicated that tdTomato-positive neurons were predominantly localized in the ipsilateral brain area, with a minority dispersed in the contralateral regions. This parallel input pattern suggests that the same upstream brain regions may target both excitatory NTS^Phox2b^ and inhibitory NTS^GABA^ neurons, presumably providing homeostatic regulation of distinct physiological functions.

### 3.3 Quantitative analysis of input neurons of NTS neurons

**Figure 6:**
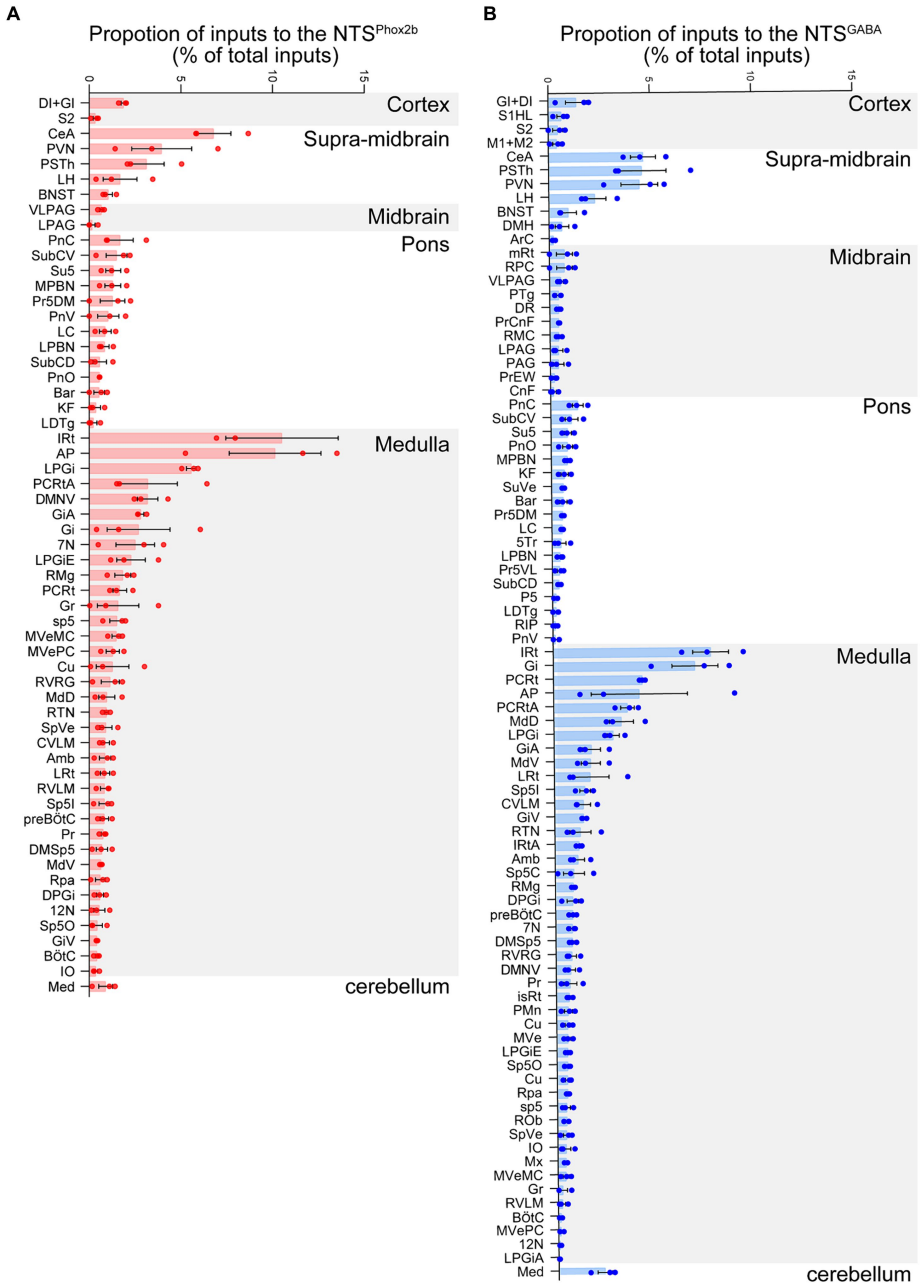
Quantitative analysis of input neurons innervating the NTS^Phox2b^ neurons and NTS^GABA^ neurons. The relative abundance of input neurons pertinent to NTS^Phox2b^ **(A)** and NTS^GABA^ **(B)** neuron populations across distinct brain nuclei was quantified by determining the ratio of tdTomato-tagged afferent neurons within each nucleus relative to the overall neuronal count.

To quantify the proportion of monosynaptic input neurons that target both NTS^Phox2b^ and NTS^GABA^ neurons, we made a statistical analysis by calculating the ratio of the number of tdTomato-labeled afferent neurons originating from each nucleus and the total number of labeled neurons in the whole brain (Figure 6, *n* = 3). The brains were divided into six structures (*n* = 86 nuclei): cortex, supra-midbrain, midbrain, pons, medulla oblongata and cerebellum. The tdTomato-labeled neurons were counted in each brain region, with each comprising >0.05% of the total labeled neurons.

As shown in Figure 6, our findings revealed that the medulla oblongata accounted for the highest quantity of inputs to the NTS, with the greatest number of inputs originating from the IRt (10.52 ± 3.07% for NTS^Phox2b^, 7.77 ± 0.88% for NTS^GABA^). Additionally, the CeA (6.79 ± 0.95% for NTS^Phox2b^, 4.66 ± 0.62% for NTS^GABA^), PVN (3.96 ± 1.64% for NTS^Phox2b^, 4.47 ± 0.90% for NTS^GABA^) and PSTh (3.13 ± 0.96% for NTS^Phox2^, 4.59 ± 1.21% for NTS^GABA^) in the supra-midbrain regions displayed heavy projections to the NTS neurons. The ventrolateral periaqueductal gray (VLPAG, 0.67 ± 0.11% for NTS^Phox2b^, 0.56 ± 0.12% for NTS^GABA^) in the midbrain, was another moderate nucleus providing inputs to NTS neurons. Conversely, the LC (0.88 ± 0.32% for NTS^Phox2b^, 0.51 ± 0.04% for NTS^GABA^), LPBN (0.85 ± 0.24% for NTS^Phox2b^, 0.40 ± 0.08% for NTS^GABA^) and Kölliker-Fuse nucleus (K-F, 0.37 ± 0.24% for NTS^Phox2b^, 0.65 ± 0.18% for NTS^GABA^) appeared to have minimal labeled neurons in the pontine nuclei.

Collectively, the findings offer a detailed whole-brain mapping of monosynaptic input neurons for both NTS^Phox2b^ and NTS^GABA^ neurons, facilitating the identification of distinct regions implicated in the modulation of various physiological processes for future investigation.

### 3.4 Mapping of efferent projections of both NTS^Phox2b^ and NTS^GABA^ neurons

**Figure 7:**
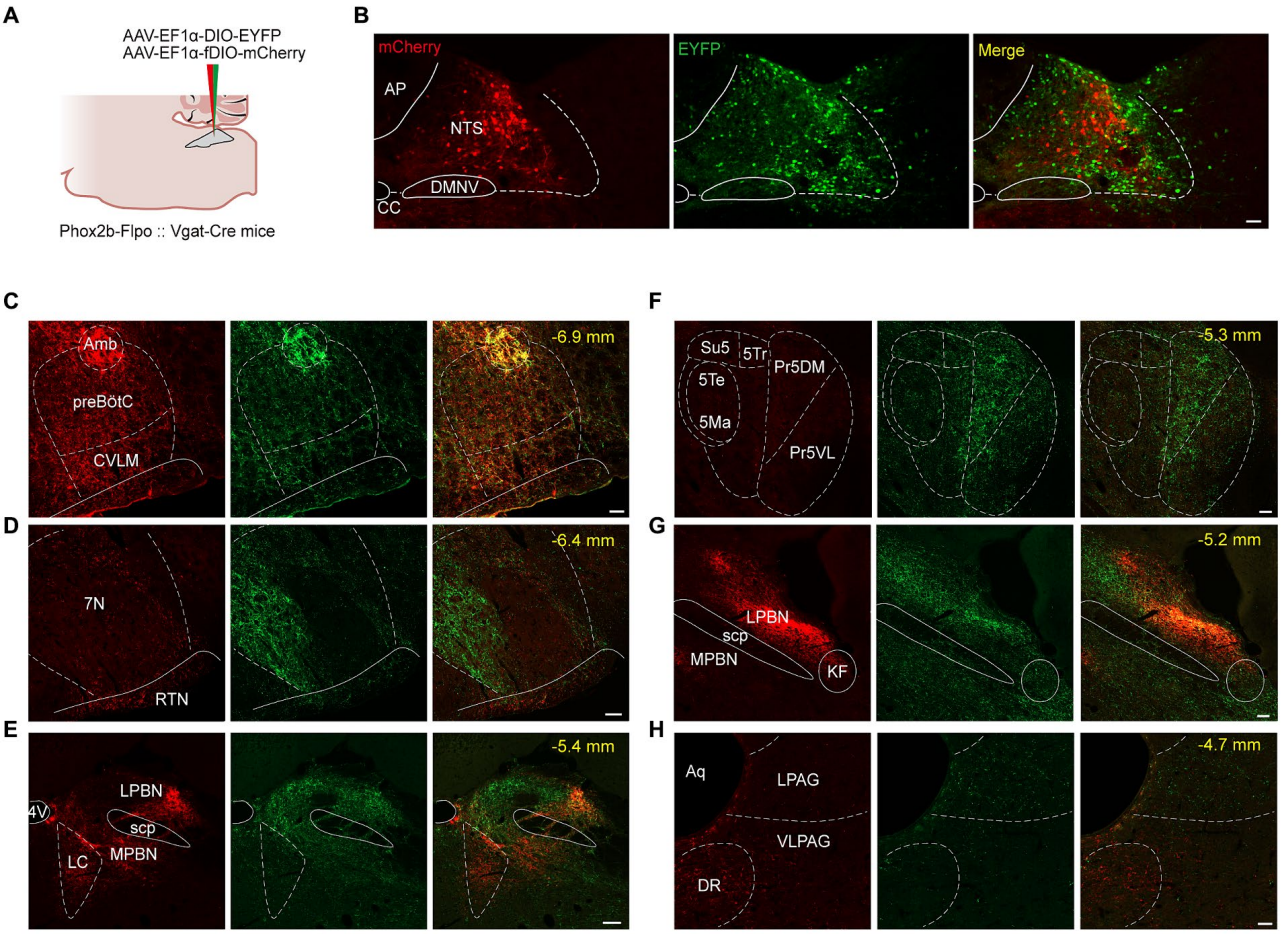
Photomicrographs of brain regions with a significant density of axon projections of NTS^Phox2b^ and NTS^GABA^ neurons in brainstem. **(A)** Schematic of viral injection strategy in PHox2b-Flpo::Vgat-Cre mice. **(B)** Images showing fluorescent NTS^Phox2b^ (mCherry) and NTS^GABA^ (EYFP) neurons. **(C–H)** Photomicrographs of parallel efferent projections of NTS^Phox2b^ and NTS^GABA^ neurons to brainstem nuclei. Scale bars: 100 μm **(B)**, 50 μm (C-H).

Next, we characterized the outputs of NTS neurons by mapping their axonal terminals, employing a widely-used anterograde viral tracing strategy as reported in previous studies (Sun et al., 2023). To concurrently visualize the axonal projections of NTS^Phox2b^ and NTS^GABA^ neurons, both AAV-EF1α-DIO-EYFP and AAV-EF1α-fDIO-mCherry were injected into the NTS from Phox2b-Flpo::Vgat-Cre mouse mice (Figure 7A). Four weeks post-injection, immunohistochemical staining revealed that both EYFP- and mCherry-expressing somata were predominantly localized within the NTS. A very small number of fluorescently labeled cells were observed in adjacent regions such as the dorsal nucleus of the vagus nerve (DMNV) and area postrema (AP) (Figure 7B). Here we presented the consistent observations of clear projection fields of both types of NTS neurons across all experiments (*n* = 3 mice).

**Figure 8:**
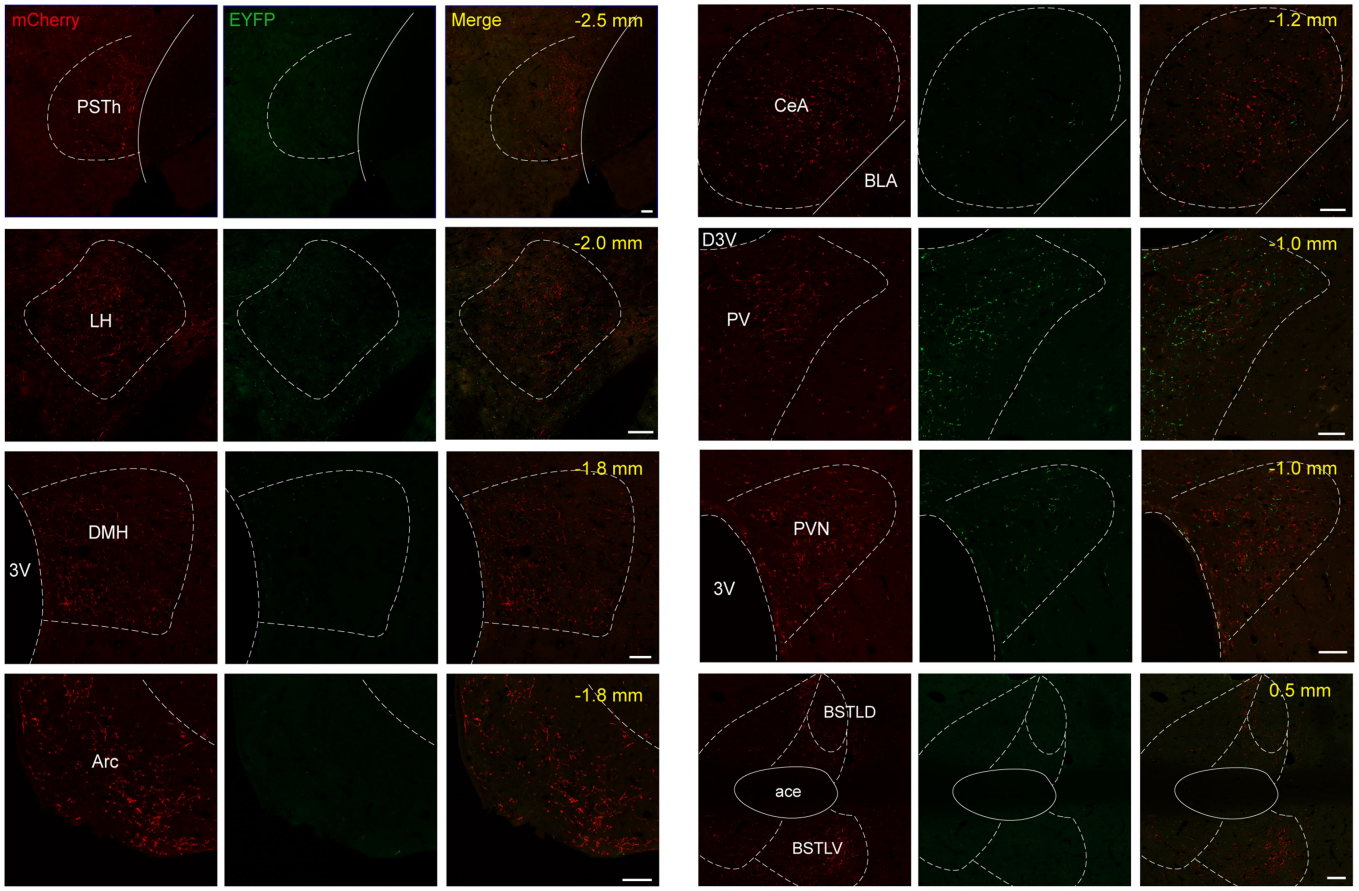
Mapping of axonal projections of NTS^Phox2b^ and NTS^GABA^ neurons in supra-midbrain areas Images showing supra-midbrain axonal projections of NTS^Phox2b^ neurons (mCherry) and NTS^GABA^ (EYFP) neurons. Scale bars, 50 μm.

Both NTS^Phox2b^ and NTS^GABA^ neurons were observed primarily projecting to the medulla oblongata, where they targeted well-established brain regions responsible for control of breathing, including the preBötC, BötC, retrotrapezoid nucleus (RTN) and facial nucleus (7 N). Additionally, projections from these neurons were also directed towards the IRt, Amb, DMNV, and hypoglossal nucleus (Figures 7C,D). The pattern of medullary projections was notably similar between NTS^Phox2b^ and NTS^GABA^ neurons. In the pons, EYFP-labeled NTS^GABA^ neurons were found projecting to the principal sensory trigeminal nucleus (Pr5DM) and the motor trigeminal nucleus (5 Ma/5Te) (Figure 7F), a distribution undetected in NTS^Phox2b^ neurons. In the midbrain, mCherry-labeled NTS^Phox2b^ neurons, rather than NTS^GABA^ neurons, specifically targeted the dorsal raphe nucleus (Figure 7H). No significant differences in axonal projections between NTS^Phox2b^ and NTS^GABA^ neurons were detected in the LPBN, K-F nucleus, LC, VLPAG, and lateral periaqueductal gray (LPAG) (Figures 7E,G,H). Region-specific projections were also observed in supra-midbrain regions. As shown in Figure 8, NTS^Phox2b^ neurons but not NTS^GABA^ neurons, extended their axons to the PSTh (Figure 8A), LH (Figure 8B), dorsomedial hypothalamic nucleus (DMH) (Figure 8C), arcuate hypothalamic nucleus (Arc) (Figure 8D), CeA (Figure 8E), and bed nucleus of the stria terminalis (BNST) (Figure 8H). Additionally, axonal terminals from both neuron types were identified in the paraventricular thalamic nucleus (PV) (Figure 8F) and PVN (Figure 8G) projections, highlighting an overlap in their projection patterns.

**Figure 9:**
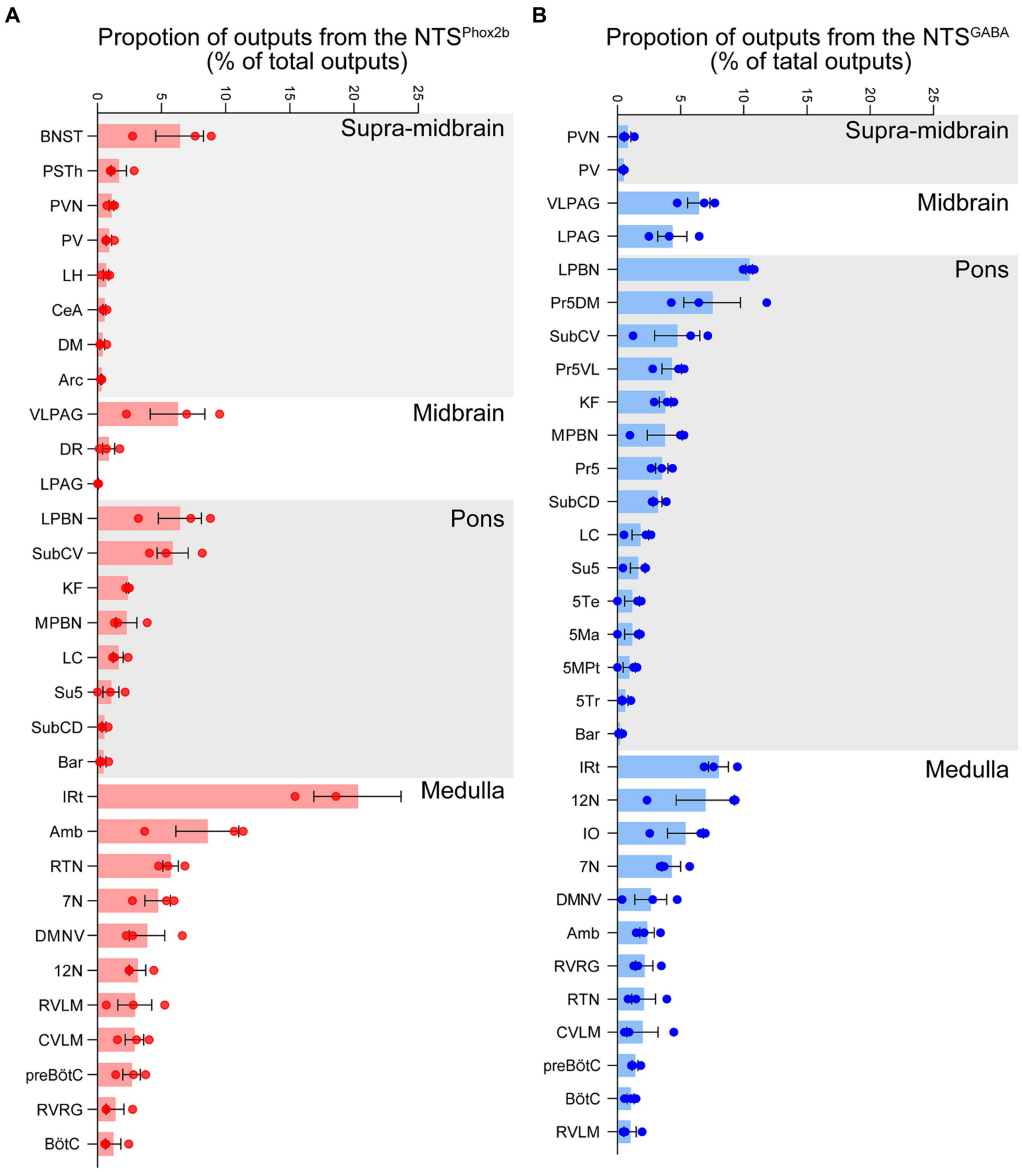
Quantitative analysis of efferent projection from NTS^Phox2b^ and NTS^GABA^ neurons. To quantify the whole-brain efferent projections of NTS^Phox2b^ **(A)** and NTS^GABA^ **(B)** neurons, the proportion of axonal projections was calculated as the ratio of the average number of varicosities to the total number of whole-brain projection varicosities.

To ascertain the projection pattern for both NTS^Phox2b^ and NTS^GABA^ neurons, we quantified pixel of axonal varicosities. Both neuron types exhibited pronounced projections within the medulla oblongata and the pons, while projections were sparser towards the supra-midbrain and midbrain, with minimal to no projections detected reaching the cortex or cerebellum. As illustrated (Figure 9A for NTS^Phox2b^, Figure 9B for NTS^GABA^, respectively), statistical analysis revealed that the dense projections from NTS neurons predominantly targeted the IRt (20.28 ± 3.40% for NTS^Phox2b^, 7.99 ± 0.79% for NTS^GABA^). Additionally, the LPBN in the pons (6.42 ± 1.68% for NTS^Phox2b^, 10.42 ± 0.26% for NTS^GABA^) and VLPAG in the midbrain (6.25 ± 2.13% for NTS^Phox2b^, 6.44 ± 0.89% for NTS^GABA^) also received some projections from both types of NTS neurons. In contrast, within the supra-midbrain regions, the BNST (6.41 ± 1.87%) and PSTh (1.66 ± 0.60%) received projections predominantly from NTS^Phox2b^ neurons rather than from NTS^GABA^ neurons, suggesting a differential projection pattern between the two neuron types.

## 4 Discussion

In this investigation, we present a comprehensive cerebral atlas that vividly delineates both the afferent and efferent projections of NTS^Phox2b^ and NTS^GABA^ neurons, representing excitatory and inhibitory neuronal types, respectively. Our findings reveal that both distinct neuronal populations not only receive monosynaptic inputs from an expansive range of cerebral territories including the medulla oblongata, pons, midbrain, supra-midbrain, and cortical areas, but also reciprocate by sending axonal outputs to the majority of these identical regions. This detailed mapping accentuates the bidirectional connectivity between the NTS and these significant brain regions. Moreover, the differential projection patterns to specific brain regions by both neuronal types implicate their pivotal roles in distinct function-specific neural circuits. This comprehensive neuroanatomical characterization enhances our understanding of the integral contributions of NTS^Phox2b^ and NTS^GABA^ neurons in modulating complex physiological functions.

### 4.1 Connection pattern of NTS^Phox2b^ and NTS^GABA^ neurons

Previous studies have utilized non-specific axon tracers to explore neural pathways associated with inputs or outputs of the NTS throughout the brain (Ricardo and Koh, 1978; Shioya and Tanaka, 1989; Herbert et al., 1990; Yu and Gordon, 1996; Tanaka et al., 1997; Rinaman, 2010; Ganchrow et al., 2014; Gasparini et al., 2020). Recently, Holt summarized a comprehensive overview of the inputs and outputs of the caudal NTS, revealing widespread inputs from six brain regions (i.e., cortex, subcortical nucleus, diencephalon, mesencephalon, pons, and medulla), along with simultaneous distribution of axonal projections and bidirectional connections among multiple nuclear regions (Holt, 2022). Built upon established neural tracing evidence, our findings provide further insight into inputs and outputs of both excitatory and inhibitory neuronal populations within the NTS, facilitating subsequent investigations aimed at examining function-specific circuits. Specifically, we have demonstrated parallel efferent projections of both NTS^Phox2b^ and NTS^GABA^ neurons within the same animals, thereby revealing the spatial distribution patterns of axonal terminals originating from these distinct neuronal populations in their respective target regions. These findings are instrumental in elucidating the circuit mechanisms underlying the differential regulation of homeostasis mediated by both neuronal populations.

We present that NTS^Phox2b^ neurons are recipients of monosynaptic inputs originating from a broad spectrum of brain regions including the medulla oblongata, pons, midbrain, supra-midbrain areas, cerebellum and cortex, with a significant density of input neurons located in the IRt, AP, CeA and PVN. Moreover, these NTS^Phox2b^ neurons project their axons towards the medulla, pons, midbrain, and supra-midbrain regions, exhibiting dense outputs particularly in the IRt, Amb, RTN, LPBN, VLPAG and BNST, while conspicuously lacking detectable projections to the cerebellum and cortex. Intriguingly, there exists an array of reciprocal connections between NTS^Phox2b^ neurons and several brain regions, predominantly including the IRt, Amb, RTN, DMNV, BNST and PSTh. Likewise, NTS^GABA^ neurons receive and dispatch monosynaptic inputs and axonal projections to the same array of brain regions as NTS^Phox2b^ neurons, with an increased prevalence of input neurons in the IRt, Gi, AP, CeA, PSTh and PVN, and more pronounced outputs in the IRt, 12 N, LPBN and VLPAG. Additionally, NTS^GABA^ neurons also establish reciprocal connections with the IRt, DMNV, AP and LPBN, illustrating complex patterns of intra- and inter-regional neural circuitries.

The quantitative assessment of synaptic inputs reveals that both NTS^Phox2b^ and NTS^GABA^ neurons receive robust monosynaptic inputs predominantly from regions located in the supra-midbrain areas such as the CeA, PVN, PSTh, and an array of medullary regions including the IRt, AP, parvicellular reticular nucleus (PCRt) and LPGi. Notably, a greater density of midbrain neurons appears to monosynaptically innervate NTS^GABA^ neurons as compared to their NTS^Phox2b^ counterparts. Furthermore, a rigorous exploration of the axonal outputs of these neuronal populations indicates that NTS^Phox2b^ neurons preferentially extend axons towards supra-midbrain nuclei like the BNST and PSTh. In contrast, the projections of NTS^GABA^ neurons are more extensively directed towards pontine regions including the Pr5DM and Pr5VL. This divergent connectivity pattern underscores the roles of NTS^Phox2b^ and NTS^GABA^ neurons in orchestrating function-specific neural circuits, which may influence various physiological processes or behaviors through distinct neural pathways.

Although we have elucidated the connectivity between the brain and NTS neurons, the present data did not encompass NTS projections to or from the spinal cord. Specifically, the descending projection to the spinal cord is derived exclusively from a small population of neurons located in the ventrolateral NTS (Saper and Stornetta, 2014). The spinal targets of this pathway include respiratory motor neurons as well as the sympathetic preganglionic column (Loewy and Burton, 1978; Dobbins and Feldman, 1994). Additionally, the NTS also receives afferents from the superficial layers of the spinal and trigeminal dorsal horns (Menetrey and Basbaum, 1987). Many of the dorsal horn neurons contributing to this pathway have neurokinin-1 receptors (Al-Khater and Todd, 2009), are activated by visceral stimuli and contain glutamate (Gamboa-Esteves et al., 2001).

In conjunction with similarities and distinctions in long-range connectivity, our observations also underline reciprocal innervation between NTS^Phox2b^ and NTS^GABA^ neurons, thereby establishing local circuits of interaction among these neuronal populations. It is noteworthy that while neurons within the NTS receive substantial afferent inputs of visceral origin, the focus of the current study is primarily centered on elucidating the central connective architecture, eschewing considerations related to peripheral inputs. This delineation allows for a more targeted analysis of intracerebral neural pathways, which contributes to a deeper understanding of the regulatory mechanisms underpinning these complex neuronal interactions.

### 4.2 Reciprocal connections of NTS with breathing-related regions in brainstem

The NTS plays an essential role in homeostatic control of breathing. In this study, we elucidate that some brainstem regions establish direct projections to both NTS^Phox2b^ and NTS^GABA^ neurons, forming recurrent circuits that modulate respiratory homeostasis. NTS neurons are integral in processing afferent signals originating from carotid body chemoreceptors, thus playing a crucial role in the modulation of peripheral respiratory chemoreflex pathways. Additionally, NTS^Phox2b^ neurons function as central respiratory chemoreceptors to provide excitatory drive to the respiratory central pattern generator (rCPG) (Fu et al., 2017, 2019). Our findings indicate that NTS^Phox2b^ neurons exhibit reciprocal projections with the RTN and LC, both acknowledged as central respiratory chemoreceptors (Wang et al., 2013a,b; Liu et al., 2021). This arrangement suggests the formation of a regulatory network of central respiratory chemoreceptors, instrumental in maintaining respiratory homeostasis. Moreover, NTS^Phox2b^ neurons establish reciprocal projection with the preBötC, BötC, LPBN and K-F nucleus, all of which contribute to rhymogenesis and pattern generation (Del Negro et al., 2018; Krohn et al., 2023). Likewise, NTS^GABA^ neurons also have bidirectional connections with putative central respiratory chemoreceptors and the rCPG as depicted above. To date, experimental data are lacked regarding control of breathing by NTS^GABA^ neurons. Takakura et al. have made initial observations indicating that a subset of NTS^GABA^ neurons interfaces with RTN chemoreceptors, mediating their inhibition in response to lung inflation, thus hinting at the complex regulatory roles these neurons may play within the respiratory control network (Takakura et al., 2007). In addition to the RTN region, pump cells of the NTS, which were first described in 2004 (Ezure and Tanaka, 2004), also projected their axons to the ipsilateral ventrolateral medulla. And axonal arborizations were found in respiration-related areas and their vicinity between the level of the retrofacial nucleus and the level a few millimeters caudal to the obex (Ezure and Tanaka, 1996).

Based on neuroanatomical evidence, activation of NTS^Phox2b^ neurons through its own sensing mechanism or synaptic inputs may provide excitatory drive to breathing, whereas stimulation of NTS^GABA^ neurons is prone to exert a brake-like effect on breathing likely through acting on central chemoreceptors or the rCPG. Of note, both types of population receive monosynaptic input arising from the preBötC, a kernel structure responsible for inspiratory rhythmogenesis. Recent findings demonstrate that in addition to inspiratory motor output, Cdh9/Dbx1-residing preBötC neurons contribute to regulating the balance between calm and arousal behaviors (Yackle et al., 2017). According to the present and previous data (Yang and Feldman, 2018; Yang et al., 2020), whether SST-expressing preBötC neurons regulate breathing pattern through a feedback circuit mechanism, for example, acting on NTS^Phox2b^ and NTS^GABA^ neurons remains unclear.

The PAG contributes to integrating motor, limbic, and sensory information to modulate behavior-related breathing, such as gasping and vocalization (Subramanian and Holstege, 2010). The stimulation of dorsal PAG and LPAG induces tachypnea and they are mostly involved in active coping strategies, such as fighting or fleeing, while the stimulation of dorsomedial PAG and VLPAG elicited bradypnea, profound respiration, dyspnea, and inspiratory apneas that it is associated with passive coping strategies, such as freezing (Gonzalez-Garcia et al., 2024). The reciprocal projections between NTS^Phox2b^ and NTS^GABA^ neurons and the PAG may reveal circuit mechanisms underlying the above respiratory effects.

### 4.3 Supra-midbrain functional input and output of NTS^Phox2b^ and NTS^GABA^ neurons

Descending inputs from the forebrain provide information regarding emotional, cognitive, and physiological state to the NTS. Thereby, the NTS neurons may orchestrate these behaviors and trigger different autonomic output, including breathing. Our findings demonstrate that the cortex project directly to both NTS^Phox2b^ and NTS^GABA^ neurons, with the putative purpose of promoting behaviors that need to be timed with certain autonomic function such as the breathing cycle, e.g., breath-hold, chewing, swallowing and vocalization.

In the current investigation, the CeA, PVN and PSTh project heavily to the NTS, in line with previous studies obtained using neural tracing dye (Gasparini et al., 2020; Kim et al., 2022; Huo et al., 2024). Previous studies applied the conventional anterograde tracing method to demonstrate the axonal connections of the NTS with the PVN (Shi et al., 2021; Liu et al., 2023), BNST (Shi et al., 2021), CeA (He et al., 2022) and Arc (Martinez de Morentin et al., 2024), a framework that was further extended using specific cell types of the NTS in the present study. The CeA is the critical centers for processing emotional behaviors, learning and fear response. The amygdala, receiving respiratory inputs, exhibits rhythmic activity that correlates with inspiratory activity signaled by the phrenic nerve root (Onimaru and Homma, 2007), suggesting that the amygdala’s spontaneous oscillatory behavior is associated with respiratory functions. In humans, electrical stimulation of the amygdala leads to an apnea (Dlouhy et al., 2015). Therefore, it appears that the CeA-NTS circuits mediate behavioral/emotional regulation of autonomic output.

The PVN plays imperative roles in the regulation of energy balance and various endocrinological activities, as well as ventilatory homeostasis (Fukushi et al., 2019). It has been shown that electrical stimulation of the PVN in anesthetized rabbits produced an increase in respiratory frequency (Duan et al., 1997); injection of glutamate into the PVN increased electromyographic activity of the diaphragm in anesthetized rats (Yeh et al., 1997). Moreover, disinhibition of the PVN with GABAA receptor antagonist increased both respiratory frequency and tidal volume in conscious rats (Schlenker et al., 2001). The most prominent role of the PVN is its involvement in the mediation of the respiratory response to hypoxia (Ruyle et al., 2019). Furthermore, the role of the PVN in mediating chemoreflex may be specific to hypoxic but not to hypercapnic stimulation (Reddy et al., 2005). However, the neural circuit mechanism through which the PVN regulates ventilation remain to be fully elucidated. The present neural tracing data may provide putative circuit mechanism underlying these effects.

### 4.4 Limitations of the study

In the present investigation, we have elucidated the whole-brain mapping of projections of both NTS^Phox2b^ and NTS^GABA^ neurons. However, the study has certain limitations due to its aim and technical challenges. First, it should be noted that this study did not examine projections to or from the spinal cord, nor did it address potential peripheral inputs to these neuronal populations. Such inputs may originate from critical regions like the carotid body and carotid sinus, as well as relay cardiopulmonary and gastrointestinal information. It stands to reason that NTS^Phox2b^ and NTS^GABA^ neurons are poised to concurrently receive both central and peripheral inputs, thereafter integrating these signals and projecting outputs to specific target neurons. Second, based on injection sites and reporter expression, NTS^Phox2b^ and NTS^GABA^ neurons were primarily distributed in the intermediate and caudal parts of the NTS (bregma −7.0 to −8.0 mm). These neurons did not extensively cover the rostral gustatory portions or the caudal commissural portion of the NTS. Third, the current study did not employ cell type-specific markers to identify target brain regions, such as the use of tyrosine hydroxylase as a marker for locus coeruleus (LC) neurons. This absence of cell type-specific markers could potentially limit the scope of quantitative analyses, particularly in the accurate enumeration of cells and axonal varicosities.

## 5 Conclusion

Collectively, we established precise anatomical connections between the specific NTS subpopulations and many brain nuclei. The whole brain mapping of inputs and outputs of both excitatory and inhibitory NTS neurons provides a foundational anatomical framework for forthcoming investigations into the inter-regional pathways involved in the respiratory functions of the NTS.

## Data availability statement

The raw data supporting the conclusions of this article will be made available by the authors, without undue reservation.

## Ethics statement

The animal study was approved by the Animal Care and Ethics Committee of Hebei Medical University. The study was conducted in accordance with the local legislation and institutional requirements.

## Author contributions

LS: Data curation, Investigation, Writing – original draft. FK: Investigation, Writing – review & editing. XT: Writing – review & editing. TD: Writing – review & editing. YW: Writing – review & editing. YJ: Writing – review & editing. XW: Writing – review & editing. HY: Writing – review & editing. FY: Writing – review & editing. CF: Methodology, Writing – original draft, Writing – review & editing. SW: Conceptualization, Supervision, Writing – original draft, Writing – review & editing.

## References

[ref1] Aklan I. Sayar Atasoy N. Yavuz Y. Ates T. Coban I. Koksalar F.. (2020). NTS catecholamine neurons mediate hypoglycemic hunger via medial hypothalamic feeding pathways. Cell Metab. 31, 313–326.e5. doi: 10.1016/j.cmet.2019.11.016, PMID: 31839488 PMC9017597

[ref2] Al-Khater K. M. Todd A. J. (2009). Collateral projections of neurons in laminae I, III, and IV of rat spinal cord to thalamus, periaqueductal gray matter, and lateral parabrachial area. J. Comp. Neurol. 515, 629–646. doi: 10.1002/cne.22081, PMID: 19496168 PMC2729698

[ref3] Brunet J. F. Pattyn A. (2002). Phox2 genes – from patterning to connectivity. Curr. Opin. Genet. Dev. 12, 435–440. doi: 10.1016/s0959-437x(02)00322-2 12100889

[ref4] Cheng W. Gonzalez I. Pan W. Tsang A. H. Adams J. Ndoka E.. (2020). Calcitonin receptor neurons in the mouse nucleus Tractus Solitarius control energy balance via the non-aversive suppression of feeding. Cell Metab. 31, 301–312.e5. doi: 10.1016/j.cmet.2019.12.012 31955990 PMC7104375

[ref5] Del Negro C. A. Funk G. D. Feldman J. L. (2018). Breathing matters. Nat. Rev. Neurosci. 19, 351–367. doi: 10.1038/s41583-018-0003-6, PMID: 29740175 PMC6636643

[ref6] Dlouhy B. J. Gehlbach B. K. Kreple C. J. Kawasaki H. Oya H. Buzza C.. (2015). Breathing inhibited when seizures spread to the amygdala and upon amygdala stimulation. J. Neurosci. 35, 10281–10289. doi: 10.1523/JNEUROSCI.0888-15.2015, PMID: 26180203 PMC4502266

[ref7] Dobbins E. G. Feldman J. L. (1994). Brainstem network controlling descending drive to phrenic motoneurons in rat. J. Comp. Neurol. 347, 64–86. doi: 10.1002/cne.903470106, PMID: 7798382

[ref8] Duan Y. F. Winters R. McCabe P. M. Green E. J. Huang Y. Schneiderman N. (1997). Cardiorespiratory components of defense reaction elicited from paraventricular nucleus. Physiol. Behav. 61, 325–330. doi: 10.1016/s0031-9384(96)00410-6, PMID: 9035265

[ref9] Dubreuil V. Ramanantsoa N. Trochet D. Vaubourg V. Amiel J. Gallego J.. (2008). A human mutation in Phox2b causes lack of CO2 chemosensitivity, fatal central apnea, and specific loss of parafacial neurons. Proc. Natl. Acad. Sci. U. S. A. 105, 1067–1072. doi: 10.1073/pnas.0709115105 18198276 PMC2242699

[ref10] Ezure K. Tanaka I. (1996). Pump neurons of the nucleus of the solitary tract project widely to the medulla. Neurosci. Lett. 215, 123–126. doi: 10.1016/0304-3940(96)12968-2, PMID: 8888011

[ref11] Ezure K. Tanaka I. (2004). GABA, in some cases together with glycine, is used as the inhibitory transmitter by pump cells in the Hering-Breuer reflex pathway of the rat. Neuroscience 127, 409–417. doi: 10.1016/j.neuroscience.2004.05.032, PMID: 15262331

[ref12] Fu C. Shi L. Wei Z. Yu H. Hao Y. Tian Y.. (2019). Activation of Phox2b-expressing neurons in the nucleus Tractus Solitarii drives breathing in mice. J. Neurosci. 39, 2837–2846. doi: 10.1523/JNEUROSCI.2048-18.2018, PMID: 30626698 PMC6462453

[ref13] Fu C. Xue J. Wang R. Chen J. Ma L. Liu Y.. (2017). Chemosensitive Phox2b-expressing neurons are crucial for hypercapnic ventilatory response in the nucleus tractus solitarius. J. Physiol. 595, 4973–4989. doi: 10.1113/JP274437, PMID: 28488367 PMC5509882

[ref14] Fukushi I. Yokota S. Okada Y. (2019). The role of the hypothalamus in modulation of respiration. Respir. Physiol. Neurobiol. 265, 172–179. doi: 10.1016/j.resp.2018.07.003, PMID: 30009993

[ref15] Gamboa-Esteves F. O. Kaye J. C. McWilliam P. N. Lima D. Batten T. F. (2001). Immunohistochemical profiles of spinal lamina I neurones retrogradely labelled from the nucleus tractus solitarii in rat suggest excitatory projections. Neuroscience 104, 523–538. doi: 10.1016/s0306-4522(01)00071-9, PMID: 11377852

[ref16] Ganchrow D. Ganchrow J. R. Cicchini V. Bartel D. L. Kaufman D. Girard D.. (2014). Nucleus of the solitary tract in the C57BL/6J mouse: subnuclear parcellation, chorda tympani nerve projections, and brainstem connections. J. Comp. Neurol. 522, 1565–1596. doi: 10.1002/cne.23484, PMID: 24151133 PMC4090073

[ref17] Gasparini S. Howland J. M. Thatcher A. J. Geerling J. C. (2020). Central afferents to the nucleus of the solitary tract in rats and mice. J. Comp. Neurol. 528, 2708–2728. doi: 10.1002/cne.24927, PMID: 32307700 PMC7942812

[ref18] Gasparini S. Resch J. M. Narayan S. V. Peltekian L. Iverson G. N. Karthik S.. (2019). Aldosterone-sensitive HSD2 neurons in mice. Brain Struct. Funct. 224, 387–417. doi: 10.1007/s00429-018-1778-y, PMID: 30343334 PMC6369013

[ref19] Geerling J. C. Engeland W. C. Kawata M. Loewy A. D. (2006). Aldosterone target neurons in the nucleus tractus solitarius drive sodium appetite. J. Neurosci. 26, 411–417. doi: 10.1523/JNEUROSCI.3115-05.2006, PMID: 16407537 PMC6674421

[ref20] Geerling J. C. Loewy A. D. (2006). Aldosterone-sensitive neurons in the nucleus of the solitary tract: efferent projections. J. Comp. Neurol. 497, 223–250. doi: 10.1002/cne.20993, PMID: 16705681

[ref21] Gonzalez-Garcia M. Carrillo-Franco L. Morales-Luque C. Dawid-Milner M. S. Lopez-Gonzalez M. V. (2024). Central autonomic mechanisms involved in the control of laryngeal activity and vocalization. Biology (Basel) 13:118. doi: 10.3390/biology13020118, PMID: 38392336 PMC10886357

[ref22] He S. Huang X. Zheng J. Zhang Y. Ruan X. (2022). An NTS-CeA projection modulates depression-like behaviors in a mouse model of chronic pain. Neurobiol. Dis. 174:105893. doi: 10.1016/j.nbd.2022.105893, PMID: 36229006

[ref23] Herbert H. Moga M. M. Saper C. B. (1990). Connections of the parabrachial nucleus with the nucleus of the solitary tract and the medullary reticular formation in the rat. J. Comp. Neurol. 293, 540–580. doi: 10.1002/cne.902930404 1691748

[ref24] Hirsch M. R. d'Autreaux F. Dymecki S. M. Brunet J. F. Goridis C. (2013). A Phox2b::FLPo transgenic mouse line suitable for intersectional genetics. Genesis 51, 506–514. doi: 10.1002/dvg.22393, PMID: 23592597 PMC4036463

[ref25] Holt M. K. (2022). The ins and outs of the caudal nucleus of the solitary tract: an overview of cellular populations and anatomical connections. J. Neuroendocrinol. 34:e13132. doi: 10.1111/jne.13132, PMID: 35509189 PMC9286632

[ref26] Huo L. Ye Z. Liu M. He Z. Huang M. Li D.. (2024). Brain circuits for retching-like behavior. Natl. Sci. Rev. 11:nwad256. doi: 10.1093/nsr/nwad256, PMID: 38288368 PMC10824557

[ref27] Jun S. Ou X. Shi L. Yu H. Deng T. Chen J.. (2023). Circuit-specific control of blood pressure by PNMT-expressing nucleus Tractus Solitarii neurons. Neurosci. Bull. 39, 1193–1209. doi: 10.1007/s12264-022-01008-3 36588135 PMC10387028

[ref28] Kang B. J. Chang D. A. Mackay D. D. West G. H. Moreira T. S. Takakura A. C.. (2007). Central nervous system distribution of the transcription factor Phox2b in the adult rat. J. Comp. Neurol. 503, 627–641. doi: 10.1002/cne.21409, PMID: 17559094

[ref29] Kim J. H. Kromm G. H. Barnhill O. K. Sperber J. Heuer L. B. Loomis S.. (2022). A discrete parasubthalamic nucleus subpopulation plays a critical role in appetite suppression. eLife 11:e75470. doi: 10.7554/eLife.75470, PMID: 35507386 PMC9119672

[ref30] Krohn F. Novello M. van der Giessen R. S. De Zeeuw C. I. Pel J. J. M. Bosman L. W. J. (2023). The integrated brain network that controls respiration. eLife 12:e83654. doi: 10.7554/eLife.83654, PMID: 36884287 PMC9995121

[ref31] Liu N. Fu C. Yu H. Wang Y. Shi L. Hao Y.. (2021). Respiratory control by Phox2b-expressing neurons in a locus Coeruleus-preBotzinger complex circuit. Neurosci. Bull. 37, 31–44. doi: 10.1007/s12264-020-00519-1, PMID: 32468398 PMC7811975

[ref32] Liu Y. Wei J. A. Luo Z. Cui J. Luo Y. Mak S. O. K.. (2023). A gut-brain axis mediates sodium appetite via gastrointestinal peptide regulation on a medulla-hypothalamic circuit. Sci. Adv. 9:eadd5330. doi: 10.1126/sciadv.add5330, PMID: 36791202 PMC9931223

[ref33] Loewy A. D. Burton H. (1978). Nuclei of the solitary tract: efferent projections to the lower brain stem and spinal cord of the cat. J. Comp. Neurol. 181, 421–449. doi: 10.1002/cne.901810211, PMID: 690272

[ref34] Martinez de Morentin P. B. Gonzalez J. A. Dowsett G. K. C. Martynova Y. Yeo G. S. H. Sylantyev S.. (2024). A brainstem to hypothalamic arcuate nucleus GABAergic circuit drives feeding. Curr. Biol. 34, 1646–1656.e4. doi: 10.1016/j.cub.2024.02.074, PMID: 38518777 PMC7617324

[ref35] Menetrey D. Basbaum A. I. (1987). Spinal and trigeminal projections to the nucleus of the solitary tract: a possible substrate for somatovisceral and viscerovisceral reflex activation. J. Comp. Neurol. 255, 439–450. doi: 10.1002/cne.902550310, PMID: 3819024

[ref36] Onimaru H. Homma I. (2007). Spontaneous oscillatory burst activity in the piriform-amygdala region and its relation to in vitro respiratory activity in newborn rats. Neuroscience 144, 387–394. doi: 10.1016/j.neuroscience.2006.09.033, PMID: 17074446

[ref37] Osakada F. Callaway E. M. (2013). Design and generation of recombinant rabies virus vectors. Nat. Protoc. 8, 1583–1601. doi: 10.1038/nprot.2013.094, PMID: 23887178 PMC4028848

[ref38] Paxinos G. Franklin K. B. J. (2013). The mouse brain in stereotaxic coordinates. San Diego: Academic Press.

[ref39] Reddy M. K. Patel K. P. Schultz H. D. (2005). Differential role of the paraventricular nucleus of the hypothalamus in modulating the sympathoexcitatory component of peripheral and central chemoreflexes. Am. J. Physiol. Regul. Integr. Comp. Physiol. 289, R789–R797. doi: 10.1152/ajpregu.00222.2005, PMID: 15919733

[ref40] Resch J. M. Fenselau H. Madara J. C. Wu C. Campbell J. N. Lyubetskaya A.. (2017). Aldosterone-sensing neurons in the NTS exhibit state-dependent pacemaker activity and drive sodium appetite via synergy with angiotensin II signaling. Neuron 96, 190–206.e7. doi: 10.1016/j.neuron.2017.09.014, PMID: 28957668 PMC5637454

[ref41] Ricardo J. A. Koh E. T. (1978). Anatomical evidence of direct projections from the nucleus of the solitary tract to the hypothalamus, amygdala, and other forebrain structures in the rat. Brain Res. 153, 1–26. doi: 10.1016/0006-8993(78)91125-3, PMID: 679038

[ref42] Rinaman L. (2010). Ascending projections from the caudal visceral nucleus of the solitary tract to brain regions involved in food intake and energy expenditure. Brain Res. 1350, 18–34. doi: 10.1016/j.brainres.2010.03.059, PMID: 20353764 PMC2909454

[ref43] Roman C. W. Derkach V. A. Palmiter R. D. (2016). Genetically and functionally defined NTS to PBN brain circuits mediating anorexia. Nat. Commun. 7:11905. doi: 10.1038/ncomms11905 27301688 PMC4912612

[ref44] Ruyle B. C. Martinez D. Heesch C. M. Kline D. D. Hasser E. M. (2019). The PVN enhances cardiorespiratory responses to acute hypoxia via input to the nTS. Am. J. Physiol. Regul. Integr. Comp. Physiol. 317, R818–R833. doi: 10.1152/ajpregu.00135.2019, PMID: 31509428 PMC6962628

[ref45] Saper C. B. Stornetta R. L. (2014). “Central autonomic system,” in The rat nervous system. (Fourth Edition). ed. G. Paxinos (Academic Press), 629–673.

[ref46] Schlenker E. Barnes L. Hansen S. Martin D. (2001). Cardiorespiratory and metabolic responses to injection of bicuculline into the hypothalamic paraventricular nucleus (PVN) of conscious rats. Brain Res. 895, 33–40. doi: 10.1016/s0006-8993(01)02011-x 11259757

[ref47] Scott M. M. Williams K. W. Rossi J. Lee C. E. Elmquist J. K. (2011). Leptin receptor expression in hindbrain Glp-1 neurons regulates food intake and energy balance in mice. J. Clin. Invest. 121, 2413–2421. doi: 10.1172/JCI43703, PMID: 21606595 PMC3104740

[ref48] Shi M. Y. Ding L. F. Guo Y. H. Cheng Y. X. Bi G. Q. Lau P. M. (2021). Long-range GABAergic projections from the nucleus of the solitary tract. Mol. Brain 14:38. doi: 10.1186/s13041-021-00751-4, PMID: 33608037 PMC7893933

[ref49] Shioya M. Tanaka J. (1989). Inputs from the nucleus of the solitary tract to subfornical organ neurons projecting to the paraventricular nucleus in the rat. Brain Res. 483, 192–195. doi: 10.1016/0006-8993(89)90054-1 2706508

[ref50] Subramanian H. H. Holstege G. (2010). Periaqueductal gray control of breathing. Adv. Exp. Med. Biol. 669, 353–358. doi: 10.1007/978-1-4419-5692-7_72 20217381

[ref51] Sun L. Zhu M. Wang M. Hao Y. Hao Y. Jing X.. (2023). Whole-brain monosynaptic inputs and outputs of leptin receptor b neurons of the nucleus tractus solitarii in mice. Brain Res. Bull. 201:110693. doi: 10.1016/j.brainresbull.2023.110693 37348822

[ref52] Takakura A. C. Moreira T. S. West G. H. Gwilt J. M. Colombari E. Stornetta R. L.. (2007). GABAergic pump cells of solitary tract nucleus innervate retrotrapezoid nucleus chemoreceptors. J. Neurophysiol. 98, 374–381. doi: 10.1152/jn.00322.2007, PMID: 17460107

[ref53] Tanaka J. Hayashi Y. Shimamune S. Nomura M. (1997). Ascending pathways from the nucleus of the solitary tract to the subfornical organ in the rat. Brain Res. 777, 237–241. doi: 10.1016/s0006-8993(97)01211-0 9449435

[ref54] Wall N. R. Wickersham I. R. Cetin A. De La Parra M. Callaway E. M. (2010). Monosynaptic circuit tracing in vivo through Cre-dependent targeting and complementation of modified rabies virus. Proc. Natl. Acad. Sci. USA 107, 21848–21853. doi: 10.1073/pnas.1011756107, PMID: 21115815 PMC3003023

[ref55] Wang S. Benamer N. Zanella S. Kumar N. N. Shi Y. Bevengut M.. (2013a). TASK-2 channels contribute to pH sensitivity of retrotrapezoid nucleus chemoreceptor neurons. J. Neurosci. 33, 16033–16044. doi: 10.1523/JNEUROSCI.2451-13.2013, PMID: 24107938 PMC3792448

[ref56] Wang Y. K. Deng T. J. Zhao X. Shao L. Q. Chen J. T. Fu C. R.. (2024). Control of breathing by orexinergic signaling in the nucleus tractus solitarii. Sci. Rep. 14:7473. doi: 10.1038/s41598-024-58075-x, PMID: 38553555 PMC10980752

[ref57] Wang S. Shi Y. Shu S. Guyenet P. G. Bayliss D. A. (2013b). Phox2b-expressing retrotrapezoid neurons are intrinsically responsive to H+ and CO2. J. Neurosci. 33, 7756–7761. doi: 10.1523/JNEUROSCI.5550-12.2013, PMID: 23637167 PMC3707793

[ref58] Yackle K. Schwarz L. A. Kam K. Sorokin J. M. Huguenard J. R. Feldman J. L.. (2017). Breathing control center neurons that promote arousal in mice. Science 355, 1411–1415. doi: 10.1126/science.aai7984, PMID: 28360327 PMC5505554

[ref59] Yang C. F. Feldman J. L. (2018). Efferent projections of excitatory and inhibitory preBotzinger complex neurons. J. Comp. Neurol. 526, 1389–1402. doi: 10.1002/cne.24415, PMID: 29473167 PMC5869167

[ref60] Yang C. F. Kim E. J. Callaway E. M. Feldman J. L. (2020). Monosynaptic projections to excitatory and inhibitory preBotzinger complex neurons. Front. Neuroanat. 14:58. doi: 10.3389/fnana.2020.00058, PMID: 33013329 PMC7507425

[ref61] Yeh E. R. Erokwu B. LaManna J. C. Haxhiu M. A. (1997). The paraventricular nucleus of the hypothalamus influences respiratory timing and activity in the rat. Neurosci. Lett. 232, 63–66. doi: 10.1016/s0304-3940(97)00579-x, PMID: 9302087

[ref62] Yu D. Gordon F. J. (1996). Anatomical evidence for a bi-neuronal pathway connecting the nucleus tractus solitarius to caudal ventrolateral medulla to rostral ventrolateral medulla in the rat. Neurosci. Lett. 205, 21–24. doi: 10.1016/0304-3940(96)12383-1, PMID: 8867011

[ref63] Yu H. Shi L. Chen J. Jun S. Hao Y. Wang S.. (2022). A neural circuit mechanism controlling breathing by leptin in the nucleus Tractus Solitarii. Neurosci. Bull. 38, 149–165. doi: 10.1007/s12264-021-00742-4, PMID: 34212297 PMC8821766

[ref64] Zhang C. Kaye J. A. Cai Z. Wang Y. Prescott S. L. Liberles S. D. (2020). Area Postrema cell types that mediate nausea-associated behaviors. Neuron 109, 461–472.e5. doi: 10.1016/j.neuron.2020.11.010, PMID: 33278342 PMC7864887

[ref65] Zhao W. Zhang K. Dong W. Y. Tang H. D. Sun J. Q. Huang J. Y.. (2024). A pharynx-to-brain axis controls pharyngeal inflammation-induced anxiety. Proc. Natl. Acad. Sci. USA 121:e2312136121. doi: 10.1073/pnas.2312136121 38446848 PMC10945766

